# High-Sensitivity Troponin: Finding a Meaningful Delta

**DOI:** 10.3390/jcdd11100318

**Published:** 2024-10-11

**Authors:** Catherine X. Wright, Donald S. Wright, Jiun-Ruey Hu, Cesia Gallegos

**Affiliations:** 1 Department of Internal Medicine, Section of Cardiovascular Medicine, Yale School of Medicine, New Haven, CT 06510, USA; 2 Department of Emergency Medicine, Yale School of Medicine, New Haven, CT 06510, USA; 3 Department of Cardiology, Smidt Heart Institute, Cedars-Sinai Medical Center, Los Angeles, CA 90048, USA

**Keywords:** high-sensitivity cardiac troponin, cardiac biomarkers, troponin delta

## Abstract

High-sensitivity cardiac troponin (hs-cTn) assays have significantly refined the resolution of biomarker-level detection and have emerged as the gold standard cardiac biomarker in evaluating myocardial injury. Since its introduction, hs-cTn has been integrated into the Fourth Universal Definition of Myocardial Infarction and various European Society of Cardiology (ESC) and American College of Cardiology/American Heart Association (ACC/AHA) guidelines for the evaluation and diagnosis of chest pain syndromes. However, despite its integral role in caring for patients with chest pain, there are still substantive gaps in our knowledge of the clinical interpretation of dynamic changes in hs-cTn values. Whether a relative or absolute hs-cTn delta should be used to detect acute myocardial injury remains debatable. There are also emerging considerations of possible sex and racial/ethnic differences in clinically significant troponin deltas. In the emergency department, there is debate about the optimal time frame to recheck hs-cTn after symptom onset for myocardial infarction rule-out and whether hs-cTn deltas should be integrated into clinical risk scores. In this review, we will provide an overview of the history of clinical utilization of cardiac biomarkers, the development of hs-cTn assays, and the ongoing search for a meaningful delta that can be clinically applicable.

## 1. Introduction

Ischemic heart disease (IHD) represents a significant burden of morbidity and mortality worldwide, and its prevalence is expected to continue to increase over time, given rising contributions from metabolic disorders and population aging [1,2]. Therefore, a safe, rapid, and accurate diagnostic method is crucial for patients suspected to have acute coronary syndromes (ACS), enabling timely initiation of evidence-based therapies and allowing for very low-risk patients to be ruled out. Over the past decades, cardiac troponin (cTn) has emerged as the gold standard cardiac biomarker in evaluating myocardial injury and infarction [3]. Subsequent technological advancements have led to the development of high-sensitivity cardiac troponin (hs-cTn) assays, which have significantly refined the resolution of biomarker-level detection, enabling the fast detection of very low troponin concentrations with high precision.

Since its introduction, hs-cTn has been integrated into and taken on a central role in both the Fourth Universal Definition of Myocardial Infarction and various European Society of Cardiology (ESC) [4] and American College of Cardiology/American Heart Association (ACC/AHA) [5] guidelines for the evaluation and diagnosis of chest pain syndromes [6,7]. Nonetheless, the need to interpret very low concentrations and minute changes in troponin concentrations may confuse many clinicians unfamiliar with this biomarker. Furthermore, there are still substantive gaps in our knowledge of what constitutes a clinically significant dynamic change in hs-cTn.

**Figure 1:**
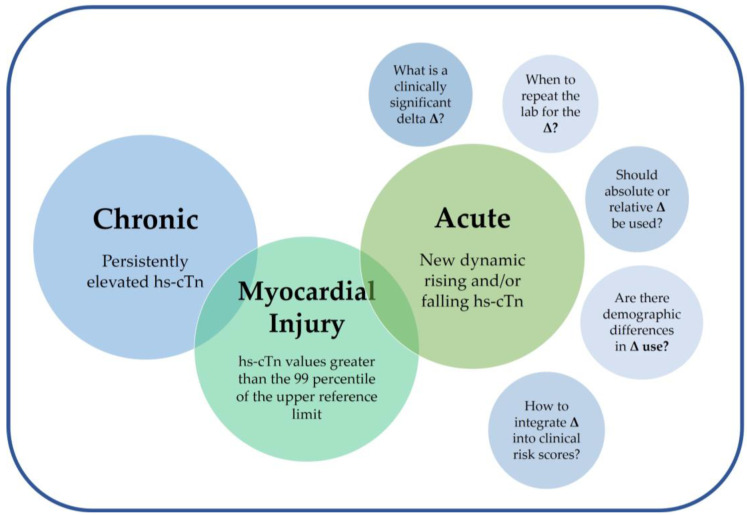
Clinical challenges in troponin delta interpretation. A summary of the definitions of acute and chronic myocardial injury, and some of the various clinical challenges associated with interpreting high-sensitivity cardiac troponin (hs-cTn) deltas.

This review will provide an overview of the history of troponin as a cardiac biomarker, the development and clinical implementation of hs-cTn assays, and the various clinical challenges associated with interpreting hs-cTn deltas (Figure 1).

## 2. The History of Troponin

In 1965, troponin, a protein component of the myofibrillar apparatus in both skeletal and cardiac myocytes, was discovered [1]. Physiologically, troponin proteins work with calcium ions to facilitate muscle contraction by regulating the interaction of actin and myosin filaments as part of the filament sliding mechanism. Cardiac troponin (cTn) is a protein complex that is made up of three subunits: troponin T (TnT), which attaches the troponin protein complex to the actin filament; troponin C (TnC), which is the calcium ion binding site; and troponin I (TnI) which acts as an inhibitor preventing interaction of the troponin protein complex with myosin heads when there is an insufficient concentration of calcium [2,3].

At rest, the troponin protein complex is attached to tropomyosin by TnT, occluding the binding site for myosin on the actin filament. TnI anchors the troponin–tropomyosin complex in place by binding it to actin [6,7]. An action potential creates cell depolarization during cardiac myocyte contraction, increasing the intracellular calcium concentration. Intracellular calcium ions bind to TnC, leading to a conformation change of the protein complex and disengagement of TnI. With both myosin and myosin-binding sites on actin filaments exposed, myosin binds to actin, producing sarcomere and, subsequently, myocardium contraction [8,9,10].

Although TnC is found in cardiac and slow skeletal muscle, cardiac isoforms of TnI and TnT are found almost exclusively in adult myocardium [6,11]. With myocardial cell damage, the systemic blood concentrations of cardiac isoforms of TnI and TnT increase. This makes TnI and TnT useful biomarkers for injury to cardiac myocytes, regardless of the cause of myocyte damage [3,8,9]. Even though there have since been studies indicating the presence of TnI and TnT in various other tissues, including skeletal muscle, it is generally accepted that TnI and TnT are the most specific biomarkers for identifying myocardial injury [3,6].

## 3. High-Sensitivity Troponin Assays

### 3.1. Pharmacokinetics of Troponin

The pharmacokinetics of cTn can be explained by the distribution of the protein in cardiomyocytes: the vast majority of cTn is found in the cardiac sarcomere as part of the troponin protein complex, while only about 4% to 5% of cTn is found as free protein in the cytoplasm [7,12,13]. Therefore, following myocardial injury, there is an initial rapid increase in serum cTn due to release from the cytoplasm, followed by a secondary serum cTn rise from the decaying myocardial contractile apparatus, which is slower, gradual, and continuous [7,9,11,12]. This is important to note as other disease processes that cause chronic myocardial injury, such as end-stage kidney disease (ESKD) or infiltrative cardiomyopathies, have different cTn cellular release kinetics, leading to chronic low-level serum cTn elevation [14]. However, as the half-life of troponin is approximately 2 h, the continuous cTn release and increase in serum concentration are highly suggestive of acute myocardial ischemia.

### 3.2. High-Sensitivity Troponin Assays

The most commonly used methods for troponin detection are immunochemical methods, such as enzyme-linked immunoassay (ELISA) or radioimmunoassay (RIA). These assays are similar: an initial immunological phase with antibody–antigen binding, a second phase of an enzymatic reaction or a secondary antibody–antigen reaction, and a final detection phase. Detection varies based on the assay method used; for instance, in ELISA, a spectrophotometer measures color intensity, while in RIA, a radiometer measures radionuclide emission. Quantification is possible as signal strength directly correlates to the amount of troponin proteins detected. Over time, several generations of troponin assays have been developed, with each iteration leading to decreases in diagnostic antibody cross-reactivity and improvements in analytical characteristics [9,15,16]. Currently, the fifth generation of troponin immunoassays represents the gold standard of cTn detection.

High-sensitivity cardiac troponin (hs-cTn) assays can detect troponins at a significantly lower serum concentration than prior assays by using antibody reagents that have considerably higher avidity for cTn proteins than prior assays, improving the overall signal-to-noise ratio for the tests [17]. It has been proposed that to qualify as a high-sensitivity assay, the assay should (1) have a total imprecision at the 99th percentile of ≤10%, and (2) for measurable concentrations of troponin below the 99th percentile, be able to attain at a concentration value above the assay’s limit of detection for at least 50% (and ideally >95%) of healthy individuals [15,18]. It has also been proposed that to decrease confusion and unnecessary decimal points and zeros, concentrations for hs-cTn assays should be expressed in nanograms per liter (picograms per milliliter) instead of the commonly published units of micrograms per liter; that is, for instance, a concentration of 0.0015 μg/L should be reported as 1.5 ng/L [18].

Given that each specific high-sensitivity troponin assay approved by the Food and Drug Administration (FDA) has different sensitivities and reference populations, the FDA recommended cutoff values; that is, the 99th percentile of the upper reference limit are assay-specific [19,20,21]. For instance, the first high-sensitivity troponin assay approved by the FDA, the Elecsys Troponin T Gen 5 Short Turnaround Time [STAT] immunoassay by Roche Diagnostics (Basel, Switzerland), has cutoff values of 22 ng/L for men and 14 ng/L for women [21], while the ARCHITECT STAT High Sensitive Troponin-I assay by Abbott Laboratories (Abbott Park, IL, USA) has a cutoff value of 26.2 ng/L [22].

## 4. Dynamic Troponin Changes and a Meaningful Delta

### 4.1. Dynamic Troponin Changes in Clinical Practice

The application of hs-cTn assays improved diagnostic sensitivity for early ACS [23,24,25]. However, this increased sensitivity, which came from being able to detect minute concentrations of cTn, is also accompanied by a decrease in diagnostic specificity for acute myocardial infarction (AMI), as many other acute or chronic disease processes can cause myocardial injury and a corresponding increase in serum cTn concentrations [25,26,27]. Although the combination of symptoms suggestive of myocardial ischemia and one or more cTn values greater than the 99 percentile of the upper reference limit is used in clinical practice for the diagnosis of AMI, the formal definition of AMI requires a dynamic change, that is, a rise and/or fall of cTn values over time [4].

Indeed, a central concept in our understanding of AMI is that the injury should lead to dynamic troponin release [4,17,21,28]. This should be accompanied by a rising or falling cTn as detected by hs-cTn assays. Furthermore, any subsequent event that leads to another phase of active myocardial damage would be able to be detected after cTn decreases from the initial insult [6,29]. If this pattern of dynamic change is not present, nonacute conditions that lead to myocardial injury, such as ESKD or compensated heart failure, are typically considered. However, it should also be noted that a dynamic change could also be accompanied by non-AMI sources of cardiac injury, including Takotsubo cardiomyopathy, cardiac amyloidosis, arrhythmia, and other systemic illnesses such as sepsis [23,25,26,30].

### 4.2. A Clinically Significant Delta

Despite the importance of a dynamic change in troponin in diagnosing AMI, there are no consensus statements or universal guidelines about what constitutes a clinically significant change in hs-cTn values. There remains debate over whether an absolute or a relative change in the hs-cTn value should be used and the optimal time interval required for the hs-cTn change to be diagnostic for AMI. Furthermore, experts suggest that the optimal criteria for determining a clinically significant hs-cTn change depends on factors including patient-specific factors and the individual hs-cTn assay used; indeed, though present guidelines do not recommend a specific threshold, individual health systems have produced their own management algorithms to guide clinicians [4,26,28].

### 4.3. Relative vs. Absolute Delta Cutoffs

Multiple studies have evaluated the utility of a relative or an absolute delta, concluding that adding a delta criterion improves overall diagnostic accuracy [18,24,27,29,31,32,33]. Apple et al. provided one of the first studies to examine delta changes in hs-cTn in 2009 [34]. This study examined the utility of percentage changes of ≥10, ≥20, and ≥30% of hs-TnI and found a ≥30% change in hs-TnI values either from baseline or follow-up optimized diagnostic specificity in patients presenting with symptoms concerning ACS [34]. A subsequent study by Eggers et al. in 2011 [23] expanded on these results by examining the utility of ≥20%, ≥50%, and ≥100% in hs-TnI values and determined that a cutoff of ≥50% would have resulted in too many false-negative results for ACS. Eggers et al. proposed that clinicians combine guidelines from the Universal Definition of AMI with a ≥20% hs-TnI delta to optimally and reliably differentiate between patients with acute and chronic causes of hs-TnI elevation.

Given the concern that a relative delta may not be optimal in specific patient populations, such as those with a high initial troponin value, subsequent studies aimed to determine if a clinically significant absolute delta exists. Reichlin et al. in 2011 [35] were among the first to evaluate this question. They found that a 2 h absolute hs-cTn change performed better than a 2 h relative hs-cTn change for accurate AMI diagnosis. Mueller et al. further studied absolute hs-cTn deltas for non-ST elevation myocardial infarctions (NSTEMI) [32]. They found that, compared to relative deltas, a ROC-optimized absolute delta generated significant added value to the discrimination of NSTEMI, mainly due to higher specificity [32].

Overall, although the hs-cTn assays used have varied across studies, a criteria of a 20% relative change in hs-cTn value or an absolute change of ≥50% of the 99th percentile value (e.g., 7 to 9 ng/L with hs-TnT depending on individual assays) have both been deemed to be reasonable definitions of a clinically significant change [27,29]. Each strategy does come with its drawbacks, with relative changes being likely to overestimate (for patients with low baseline values) or underestimate (for patients with high baseline values) dynamic cTn changes, while absolute changes are dependent on the specific hs-cTn assay being used and the individual patient (individual clinical risk factors, baseline troponin values, biological availability, etc.).

**Figure 2:**
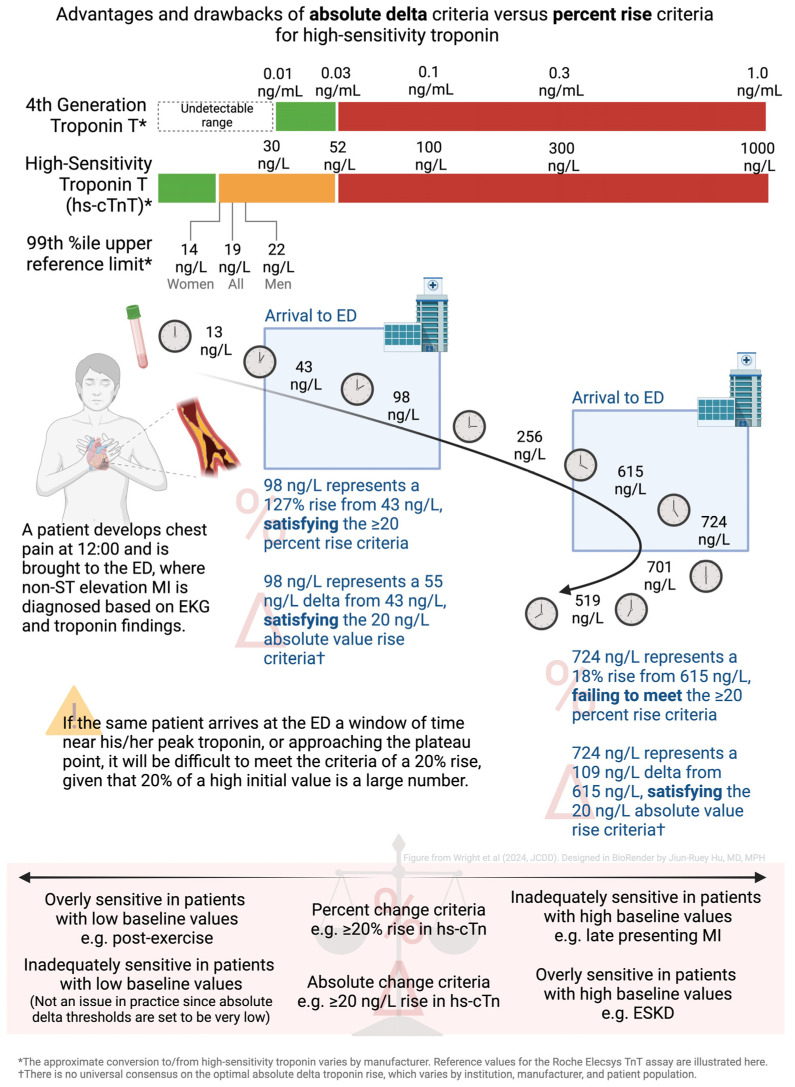
Advantages and drawbacks of absolute delta criteria versus percent rise criteria for high-sensitivity troponin. High-sensitivity serum troponin values were previously below the limit of detection in earlier generation assays. There has been a shift to using absolute delta criteria instead of percent rise criteria. For a given patient who develops chest pain at 12:00, depending on the window of time along his/her presentation to the ED, the progression from the first to second measured values of serum troponin may meet the absolute value criteria but not meet the percent rise criteria. Abbreviations: %ile: percentile; ED: emergency department; ESKD: end-stage kidney disease; hs-cTn: high-sensitivity cardiac troponin; MI: myocardial infarction.

Some experts suggest that absolute delta thresholds may be of greater utility in the era of hs-cTn, particularly as an absolute delta performs well in patients with very low baseline hs-cTn (possibility of large relative delta with a minimal absolute delta) or high initial hs-cTn (such as in patients with late presenting ACS, the possibility of small relative delta with a large absolute delta) [25,36,37]. A clinical scenario where absolute delta could be favored over a relative delta is discussed in Figure 2. Other experts suggest using an absolute change criterion in patients with a baseline hs-cTn value ≤ 99th percentile and a relative change criterion in patients with a baseline hs-cTn value > 99th percentile [29]. Furthermore, due to the inherent variability that is introduced when different commercially available FDA-approved hs-cTn assays are used in different health systems, many individual health systems have now produced their own hs-cTn interpretation algorithms to guide clinicians [4,26,28]. Clinicians should refer to their health system’s specific guidelines and algorithms if available.

### 4.4. Sex- and Race-Specific Delta Cutoffs

Women have lower baseline troponin values than men in healthy populations, which is possibly partly explained by biological differences such as lower left ventricular mass [38,39]. In fact, multiple guideline groups have recommended sex-specific 99th percentiles for clinical practice, and all FDA-approved hs-cTn assays report individualized sex-specific 99th percentiles [4,28]. All these aim to combat underdiagnoses of cardiac chest pain and AMI in women [5,28]. Further studies on recommended sex-specific 99th percentiles’ effect on clinical practice and outcomes are ongoing [40]. However, current clinical algorithms have not proposed sex-specific deltas [41], though individual studies have suggested their utility [42]. For instance, a 2020 study by Kimenai et al. [43] showed that using sex-specific hs-cTn thresholds improved ACS diagnosis. A study by Liu et al. in 2022 also found that for AMI rule-ins, sex-specific delta thresholds based on 90% specificity (14 ng/L for males, 11 ng/L for females) performed well [42]. However, no significant impact on clinical management and prognosis was seen [43].

Much less has been studied on racial and ethnic differences in cardiac biomarkers, including cTn. Several studies have suggested that Black individuals have higher baseline and 99th percentile troponin levels than non-Black individuals [11,44,45]. However, there are currently no FDA recommendations or consensus guidelines recommending race-specific cTn cutoffs.

### 4.5. Timing the Troponin Delta

Just as elevated hs-cTn values and clinically significant dynamic hs-cTn value changes are essential in diagnosing ACS, negative hs-cTn values and changes are equally important in ruling out cardiac chest pain. This is especially important given that emergency department (ED) volume and boarding times are constantly increasing, and timely workup and management are vital for the more than 20 million people who present to the ED to be evaluated for ACS annually [46,47].

For patients who present late, defined in the literature as presenting more than 2 h after symptom onset (more than 3 h in the 2021 AHA/ACC guidelines [5]), multiple studies have shown that a single low hs-cTn value can identify patients with low risk of AMI [28,48,49,50]. These patients have been followed by longitudinal studies and were found to be unlikely to experience major adverse cardiovascular events during both short- and long-term follow-up, and therefore can be considered low-risk and be discharged early from the ED [28,46,51]. However, it must be noted there is no universal definition for a low level of troponin, which could refer to the LoD, the limit of quantification (LoQ), or an assay-specific validated hs-cTn value that balances sensitivity and specificity: currently, guideline recommendations are based on the LoD value [5,28,52]. It is important to note that hs-cTn assays approved by the FDA to be used in the United States (US) only report the LoQ, not the LoD [18]. This issue is because the LoQ values are almost always higher than the LoD, though the difference may be negligible depending on the individual hs-cTn assay [53]. Although the ACC/AHA recommendation for identifying low-risk patients uses LoD values, it is clinically challenging to apply in the US. Nonetheless, studies have shown that the LoQ value performs well in identifying patients with chest pain at low risk for AMI and that a single hs-cTn LoQ value of <6 ng/L is also promising [49,54,55].

Given that the accuracy of AMI diagnosis is increased with analysis of a rising or falling pattern in hs-cTn, several algorithms for repeating troponin to rule out AMI have been evaluated [28,51,56,57,58,59]. As hs-cTn assays have been in use outside of the US for more than a decade before clinical adoption in the US, the ESC has provided rapid AMI rule-out protocols since 2011. In 2011, a 0 and 3 (0/3 h) hour rapid-rule-out protocol with the use of hs-cTn was recommended [60], with the subsequent addition of a 0 and 1 (0/1 h) hour rule-out protocol in the 2015 guidelines, both as Class I recommendations [61]. In the most recent ESC guidelines from 2020, however, the 0/3 h hour protocol has been downgraded to a Class II recommendation, with the 0/1 h or the 0 and 2 h (0/2 h) ESC algorithms recommended as Class I recommendations instead [41], as there have now been multiple studies showing that the 0/3 h ESC algorithm has a decreased sensitivity and negative predictive values for AMI rule-out as compared to the 0/1 h or 0/2 h algorithms [58,62].

However, it should be noted that the latest AHA/ACC guidelines from 2021 do not differentiate between these algorithms and recommend repeat hs-cTn sampling at 1, 2, or 3 h from ED arrival, which can be considered for AMI rule-out [5]. Interestingly, most recently, clinical policies from the American College of Emergency Physicians (ACEP) from 2018 have suggested using a 0/2 h algorithm to identify low-risk patients for more rapid discharge from the ED [63]. Studies have also proposed that strict time intervals may not be necessary for hs-cTn in evaluating ACS; that is, hs-cTn “velocity” may be just as valid as hs-cTn deltas [64]. Therefore, an optimal algorithm for timing to repeat hs-cTn to rule out AMI remains to be determined.

### 4.6. Integration of Clinical Risk Scores with Troponin Delta

Given the importance and prevalence of ACS workup in the ED, several EDs have evaluated the utility of combining clinical risk scores with hs-cTn deltas. Although some studies have found positive impacts of combining hs-cTn deltas pathways with the HEART score [65] on optimizing risk stratification [66] or reducing admission rates [67], results have been mixed with other studies showing no improved classification performance for AMI diagnosis [68]. Some possible explanations for these discrepancies include different underlying risk profiles in the studied populations [68]. There have also been concerns that the clinical risk scores, such as the HEART score, may require recalibration in the era of hs-cTn [69,70]. Currently, this is still an area of active research, and the incorporation of clinical risk scores with hs-cTn delta algorithms has not been endorsed by any major society guidelines.

## 5. Conclusions

Since its introduction, hs-cTn has been integral in the workup and management of patients with chest pain. However, there are still substantial gaps in our knowledge of the clinical interpretation of hs-cTn values. Further studies on the contribution of sex, race, and ethnicity to normal baseline hs-cTn values and clinically significant hs-cTn deltas will add to our understanding and application of this biomarker. The debate remains around what constitutes a clinically significant relative or absolute hs-cTn value change to diagnose AMI and the optimal timing to repeat hs-cTn to optimize test sensitivity and specificity for ruling out AMI in patients with chest pain.

## Data Availability

No new data were created or analyzed in this study. Data sharing is not applicable to this article.
